# Nuclear factor of activated T cells as a driver of tumor progression and a target for precision therapy

**DOI:** 10.1002/ame2.70251

**Published:** 2026-07-01

**Authors:** Hira Khan, Khushbakhat Alia, Yusra Al Dhaheri, Muhammad Naseem, Rabah Iratni, Edgar Serfling, Khalid Muhammad

**Affiliations:** ^1^ Department of Biology, College of Science United Arab Emirates University Al Ain UAE; ^2^ Department of Environmental Science and Sustainability, College of Natural and Health Sciences Zayed University Abu Dhabi UAE; ^3^ Department of Bioinformatics, Biozentrum Am Hubland University of Würzburg Würzburg Germany; ^4^ Department of Pathology University of Würzburg Würzburg Germany; ^5^ Zayed Bin Sultan Al Nahyan Center for Health Sciences United Arab Emirates University Al Ain UAE

**Keywords:** cancer progression, immune evasion, nuclear factor of activated T cells (NFAT), precision cancer therapy, tumor microenvironment (TME)

## Abstract

The Nuclear Factor of Activated T Cells (NFAT) family comprises closely related transcription factors. Numerous biological processes including angiogenesis, invasion, migration, proliferation, and cell survival are regulated by the NFAT family. NFATs are overexpressed and have increased transcriptional activity in a variety of human solid tumors and hematological cancers. Beyond tumor cell‐intrinsic roles, NFAT has also emerged as an important regulator of the tumor microenvironment (TME), where it can influence immune cell behavior and contribute to mechanisms of immune evasion. The discovery of the multifaceted functions of NFATs has driven the need to further unveil their role in cancer and provide new insights into other potential roles. This review provides a comprehensive narrative synthesis of current molecular and clinical studies, with particular emphasis on how NFAT shapes tumor immune interactions and modulates the TME. By integrating findings across different cancer types, we highlight how NFAT may contribute to both tumor progression and immune regulation. The review concludes by highlighting significant knowledge gaps and recommending future paths for translational and therapeutic research to leverage NFAT signaling as a potential target in precision cancer therapy.

## Graphical abstract

Overview illustration of dysregulated NFAT activation coordinating upstream signaling, post‐translational regulation and cancer promoting pathways to induce abnormal gene expression that drives tumor development, progression and immune escape.

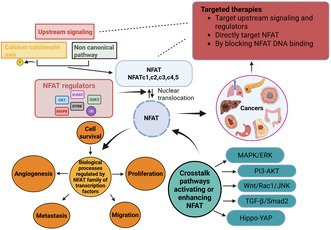

## 1 INTRODUCTION

Nuclear factor of activated T cells, commonly termed NFAT, was originally recognized as nuclear protein that binds upon activation of human and murine T cells to the interleukin 2 (IL2) promoter/enhancer.^1^, ^2^ Later experiments showed that this DNA‐binding activity is cyclosporin A (CsA) sensitive, which selectively reduces binding to the purine‐rich regions of the IL2 promoter. These findings provided key insights into the immunosuppressive action of CsA.^3^ Although first described as the Pu‐box factor due to its presence in B cells, NFAT was later renamed by Crabtree to highlight its role in activated T cells.^2^, ^3^, ^4^, ^5^ CsA, a compound derived from fungi, became widely used as an immunosuppressant, even before its exact mechanism was understood.^6^, ^7^, ^8^ CsA dampens T‐cell activation by interfering with calcium signaling, blocking calcineurin and preventing NFAT from activating IL‐2 expression.^9^, ^10^, ^11^

The first NFAT protein (NF‐ATp) was cloned in 1993 by Anjana Rao's laboratory and shown to regulate IL‐2 expression in T cells through interaction with AP‐1, while a related factor, NFATc, was later identified as a key integrator of calcium and protein kinase C signaling^12^, ^13^, ^14^, ^15^, ^16^ The family of NFATs consists of 5 members with 4 calcium‐regulated genes (*NFATc1–NFATc4*) and a single osmotic stress‐responsive gene (*NFAT5*).^17^, ^18^, ^19^ Activation happens by intracellular calcium influx via either the PLC‐γ or store operated calcium channels, with the exception of NFAT5. Hyperphosphorylated variants of the calcium‐responsive NFAT proteins are found in the cytoplasm and are activated by the rise of intracellular calcium levels, which causes calcineurin to dephosphorylate NFAT and thus leading to translocation to the nucleus.^20^, ^21^ Once in the nucleus, they bind directly to DNA and often cooperate with transcription partners like AP‐1 to mediate the transcription of downstream gene targets.^22^, ^23^, ^24^

NFAT family members are increasingly recognized for their involvement in a wide range of cellular processes, including cell fate determination, proliferation, differentiation, and development.^25^ In line with these roles, emerging evidence suggests that dysregulated NFAT signaling may contribute to tumor‐related processes such as angiogenesis, cell motility, and cell cycle progression.^23^ Numerous cancer types, such as diffuse large B cell lymphoma,^26^, ^27^ aggressive T cell lymphoma,^28^ pancreatic cancer,^29^ breast cancer,^30^, ^31^ and Burkitt's lymphoma, have also been shown to overexpress or constitutively express NFAT family members. Along with the elevated protein concentration of the NFAT family members, the NFAT gene aberration has also been found.^22^

With the growing evidence of NFAT involvement in both cancer cell intrinsic pathways and environmental pathways affecting tumor progression and therapeutic response, a systematic synthesis of NFAT biology in cancer is timely and necessary. Furthermore, advances in technologies such as single‐cell and spatial transcriptomics can provide deeper insights into the context‐specific functions of NFAT isoforms within tumors. While these findings highlight the potential relevance of NFAT signaling in cancer, its suitability as a therapeutic target remains to be fully established. This review therefore aims to summarize current knowledge and explore future directions in understanding NFAT signaling in cancer.

## 2 NFAT FAMILY STRUCTURE AND MOLECULAR REGULATION

The five members of the NFAT gene family in humans are *NFATc1* (NFAT2/NFATc), *NFATc2* (NFAT1/NFATp), *NFATc3* (NFAT4/NFATx), *NFATc4* (NFAT3), and *NFAT5* (TonEBP/OREBP).^32^

### 2.1 Diversity and domain architecture of NFAT proteins

**FIGURE 1:**
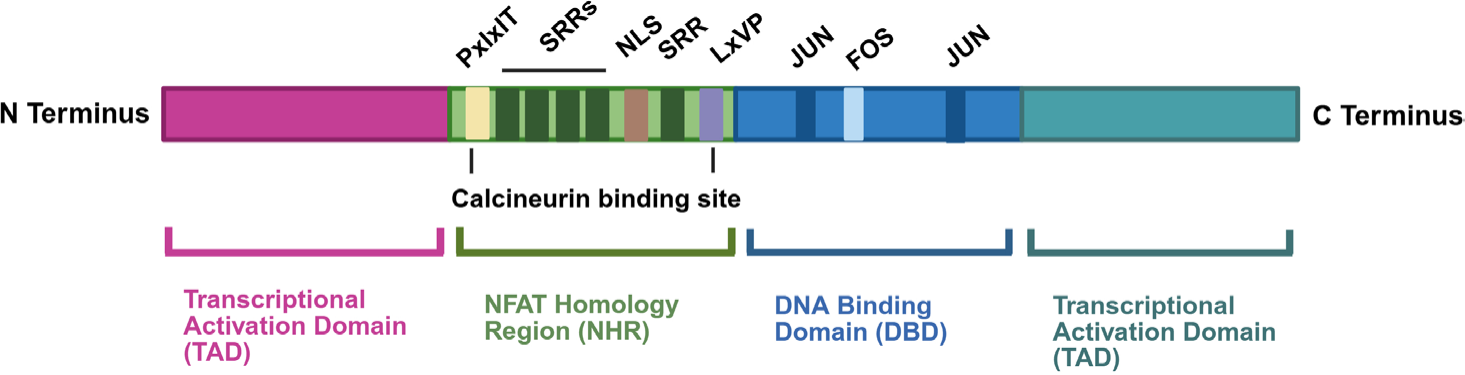
NFAT protein structure showing the NHR regulatory region containing calcineurin‐binding motifs, the DNA‐interacting DBD and the flanking TAD‐N and TAD‐C regions.

The NFAT family proteins are regulated by calcium and have a conserved modular structure consisting of two large domains in tandem: (1) a regulatory domain, also called the NFAT homology region (NHR), and (2) a DNA‐binding domain (DBD), also called the Rel homology region (shown in Figure 1). Over a stretch of approximately 300 amino acid residues this factor showed more than 70% sequence similarity to NF‐ATp and the cloning of two further NFAT factors, designated as NF‐AT3 and NF‐AT4 (or NF‐ATx),^33^, ^34^ revealed the other two genuine members of NFAT factor family. Although this domain shares less than 20% sequence similarity with the Rel DNA binding domain of NF‐kB factors, its function as a DNA binding domain and organization in two loops and 10β strands at positions similar to the Rel domain^35^ led to its designation as Rel Similarity (or Rel Homology) Domain, RSD (or RHD). The RSD mediates the binding of NFATs to their canonical ‘core’ binding DNA motif A/T GGAAA. In contrast to the RSD, the other parts of NFATs are less well conserved among the four factors. However, short conserved motifs for the interaction with Calcineurin, with the co‐factor CBP/p300, for nuclear import and export, phosphorylation and sumoylation have been identified in stretches near the 5′ and 3′ ends of both *NFATc1*, *NFATc2* and the other NFAT proteins. Those sequences located outside of RSD domains exhibited a strong transactivation activity when tested in co‐transfection studies.^36^ Conservation of the NHR is comparatively low, with only 22–36 sequence identities between various NFAT members. Two transcriptional activation domains (TAD) at the N‐ and C terminus are located on either side of the NHR and DBD domains. TADs differed greatly among NFAT's many isoforms and members.^32^, ^37^, ^38^

TADs are anticipated to be regions where particular NFAT proteins interact with particular domains to regulate essential functions like cell death and proliferation. The N‐terminal motif, central motif, and C‐terminal motif are three highly conserved small motifs found in TADs, despite being the least conserved members of the NFAT family. It is hypothesized that these conserved sub‐regions provide the structural or functional anchoring sites required for specific interactions between proteins.^39^ Through their conserved DBD and NHR domains, NFATs can interact with other proteins to either synergize^40^, ^41^ (like GATA‐3^42^; ICSBP^43^; C/EBP^44^; AP‐1^40^) or repress (PPAR‐γ^45^; Mrj^46^; Foxp3^47^; ICER^48^) NFAT‐mediated transcription.^49^

### 2.2 Canonical calcium–calcineurin‐dependent activation

The NFAT factors are normally inactive in the cytosol of unstimulated cells. Numerous cell surface receptor types, including G‐protein‐coupled receptors (GPCRs), T‐cell receptors (TCRs), and receptor tyrosine kinases (RTKs), stimulate these proteins in the cytoplasm. Actually, when these receptors are engaged by binding to their ligands, phospholipase Cγ (PLCγ) is activated, which causes phosphatidylinositol 4,5‐bisphosphate (PIP2) to be cleaved into inositol‐1,4,5‐triphosphate (IP3) and diacylglycerol (DAG). Then, IP3 joins forces with IP3 receptors that are attached to the ER to help release Ca^2+^ from ER Ca^2+^ storage. Store‐operated calcium channels (SOC) detect the ensuing Ca^2+^ depletion and cause these channels to open, allowing extracellular Ca^2+^ to enter the cytosol.^50^, ^51^ Ca^2+^ activates the Ca^2+^ sensor calmodulin (CaM) in the cytosol, which binds to and activates calcineurin (calcium‐binding protein CnA and CnB subunits). NFAT nuclear translocation is interrupted by the known phosphatase calcineurin which dephosphorylates multiple phosphoserines of NFATs. NFAT interacts with several transcription factors (e.g. ATF2, c‐Jun, c‐Fos, MEF2 and GATA4) in the nucleus in order to regulate numerous gene expression programmes.^22^, ^37^ In addition, within the nucleus, the NFAT proteins are rephosphorylated and inactivated by various kinases like casein kinase 1 [CK1], glycogen‐synthase kinase 3 [GSK3], Jun N‐terminal kinase [JNK2] and dual‐specificity tyrosine‐phosphorylation‐regulated kinase1/2 [DYRK1 and DYRK2] and move to the cytoplasm.^52^, ^53^, ^54^

### 2.3 Non‐canonical regulatory pathways governing NFAT activity

The more complex molecular activation of *NFAT5* influences its definition. Under isotonic conditions regulated by tonicity stress, *NFAT5* is constantly transferred between the cytoplasm and the nucleus. A hypertonic environment triggers *NFAT5* transcription, translation, and nuclear importation, whereas the hypotonic environment triggers nuclear export of the proteins.^55^, ^56^ *NFAT5* is turned on by diverse isotonic stimuli, including activation triggers of innate immunity receptors such as Toll‐like receptors (TLR), followed by the activation of reactive oxygen species (ROS) and mitogen‐activated protein kinases (MAPK). Interestingly, *NFAT5* has distinct characteristics in relation to osmotic activated response.^57^ However, there is still uncertainty regarding the exact biochemical pathways and the connection between these two activation mechanisms.^58^

Besides the classical calcium–calcineurin pathway, NFAT can be activated by a non‐conventional mechanism in early thymocytes. In thymocytes with pre‐TCR, *NFATc1* is activated by IL‐7‐Jak3 signaling without calcineurin‐mediated dephosphorylation and interacts with STAT5 to control survival and differentiation programs, including the stimulation of transcription of anti‐apoptotic genes, such as Bcl‐2. These results highlight the fact that there are context‐dependent NFAT activation pathways involved in T‐cell differentiation and immune homeostasis.^59^

### 2.4 Post‐translational modifications

In the cytosol of resting cells, NFAT is extensively phosphorylated at numerous Ser‐rich regions (SRR) and Ser‐Pro (SP) sequence motifs. An escalated amount of calcium results in the loss of these phosphate groups by the phosphatase calcineurin which exposes nuclear localization signals permitting the entry of NFAT into the nucleus to activate gene transcription. NFAT can be re‐phosphorylated by certain kinases to prevent this activity and it is re‐imported into the cytoplasm and silenced. A set of serine/threonine kinases specifically regulate the movement and activity of NFAT, such as GSK‐3 that phosphorylates NFAT upon priming by protein kinase A (PKA)^60^ and which is in turn inhibited by PI3K‐Akt signaling, which is one of the most commonly deregulated cancer pathways.^61^ NFAT is also regulated by other kinases, such as CK1 and MAPKs by phosphorylation of various motifs, which connects NFAT to cancer‐related signaling pathways.^62^, ^63^ Members of the DYRK family were found to be the key regulatory factors of NFAT. DYRK1 facilitates the export of NFAT out of the nucleus and DYRK2 plays a context‐dependent role both as tumor suppressor and an oncogene, making it a promising therapeutic target.^64^, ^65^ NFAT activation can also be controlled by other post‐translational modifications in addition to phosphorylation, such as sumoylation, which facilitates retention in the nucleus, and ubiquitination. A combination of these calcium‐dependent and kinase‐mediated actions indicates that the NFAT regulation is quite complex and is linked to significant cancer‐signaling pathways, which is why it is essential to regulate the cell growth and tumor progression.^23^

## 3 NFAT SIGNALING IN CANCER PATHOGENESIS

Numerous cellular processes, including angiogenesis, invasion, migration, proliferation, and survival are regulated by the NFAT family of transcription factors. Many cancer types exhibit constitutively active and elevated NFAT isoforms, which drive the transcription of downstream targets important to the development and progression of cancer. Despite the fact that a key role is played by the NFAT family in the prognosis of cancer, distinct isoforms have different functions in various cellular contexts.^22^

### 3.1 Transcriptional targets driving proliferation, invasion and angiogenesis

Evidence from in vitro and in vivo studies shows that NFAT regulates the expression of pro‐invasive (TWIST1, AQP5), angiogenic mediator (VEGF, 5‐catenin) and survival regulator (MDM2, c‐Myc) genes through *NFATc1, NFATc2, NFAT5* etc.^22^ Collectively these findings mentioned in Table 1 suggest that NFATs are an essential transcriptional center of interaction between calcium signaling and tumor pathogenesis and a promising oncolytic therapeutic target.

**TABLE 1:** Comparative summary of the activity of NFAT proteins and downstream pathways contributing to tumor progression.

NFAT members	Cancer type /Model	Key transcriptional targets/Pathways	Proposed mechanism	Biological outcome	Supporting evidence and findings
*NFATc1*	Colorectal carcinoma (Stage II–III, human + murine models)	TWIST1, ANGPTL2, COL3A1, FAP, MRC2, PTRF	Overexpression activates invasion/ECM remodeling; knockdown suppresses metastasis	Increased invasion, metastasis and angiogenesis	Integrated patient data and functional assays confirm *NFATc1*‐driven metastatic gene program^66^
	Glioblastoma (MES subtype)	HDAC1/NF‐κB axis	*NFATc1* upregulates HDAC1 maintaining NF‐κB activity	Increased tumor growth, mesenchymal transition and angiogenesis	*NFATc1* knockout reduces GSC growth; HDAC1 rescue restores phenotype^32^, ^67^
*NFATc2*	Breast carcinoma	MDM2/p53 axis	NFAT1 activates MDM2, suppressing p53‐dependent apoptosis	Increased survival and angiogenesis	NFAT1 correlates with MDM2 and vascular markers^68^
	Colorectal cancer (CRC cell lines, in vivo liver metastasis models)	ASIC2 activates Calcineurin/*NFATc2* pathway	Acid exposure induces ASIC2 activates invasion associated genes	Increased proliferation, invasion and liver metastasis	CsA blocks *NFATc2* activation and suppresses CRC metastasis^69^
	CRC stemness (CCSCs)	Hippo–YAP1 pathway	*NFATc2* reduces YAP phosphorylation, increasing nuclear YAP activity	Increased CSC stemness and tumor aggressiveness	*NFATc2* silencing decreases YAP activity and reduces CSC functions^70^
	Glioblastoma (In vitro: U87 cells, patient derived GSCs)	IL‐6/STAT3 and NDEL1/ERK axes	Promotes IL‐6 and NDEL1 transcription sustaining STAT3 and ERK activation	Increased invasion, stemness and tumor growth	*NFATc2* knockdown or calcineurin inhibition reduces invasiveness^32^
	Lung adenocarcinoma (in vitro and in vivo)	SOX2, ALDHA1, Ca^2+^/*NFATc2* axis	*NFATc2* upregulates SOX2, suppresses ALDHA1, and promotes lung CSC self‐renewal, motility, and tumor initiation	Increased invasion, migration and proliferation	High *NFATc2* in lung tumors correlates with metastasis, knockdown suppresses motility & proliferation^71^
*NFATc3*	Breast cancer (in vitro breast cancer cell lines, HUVECs, xenograft tumor models)	VEGF, SFRP2, Notch1/Orai1/SOCE	*NFATc3* mediates VEGF angiogenesis, SFRP2 tubulogenesis, and Notch1/Orai1‐driven metastasis in breast cancer	Increased angiogenesis, invasion, and tumor growth	Tacrolimus and QDG inhibit *NFATc3*, reducing breast cancer angiogenesis and tumor growth^32^
*NFAT5*	CRC stemness/dedifferentiation (ETBF‐treated colon epithelial models)	JMJD2B, NANOG	NFAT5 activates JMJD2B, which demethylates the NANOG promoter, leading to increased NANOG expression	Increased NANOG, CSC phenotype and tumorigenesis	ETBF‐induced TLR4 activation increases *NFAT5/*JMJD2B/NANOG axis^72^
	Non‐small‐cell lung carcinoma (A549, H1975)	Aquaporin‐5 (AQP5)	*NFAT5* transcriptionally activates AQP5 driving EMT and migration	Increased proliferation, migration and metastasis	*NFAT5* knockdown or UNBS1450 reduces proliferation and migration^73^
	Breast cancer (inflammatory subtype, clinical samples and in‐vitro)	β‐Catenin/CTNNB1 axis, CDH1	*NFAT5* interacts with β‐catenin and MGA enhancing angiogenesis	Increased aggressiveness, angiogenesis and survival	High *NFAT5* IHC and NFAT–CDH1 axis validated by siRNA assays^74^
*NFATc1* & *NFATc2*	Aggressive B‐cell lymphomas (DLBCL, MCL, Burkitt)	CD40L/CD154, BLyS, c‐Myc, IL‐10/STAT3, NF‐kB co‐activation	NFAT persistently activates with NF‐kB to induce CD40L, BLyS, c‐Myc, and IL‐10/STAT3 survival pathways	Increased survival, proliferation, lymphoproliferation and Immune evasion	Nuclear NFAT2 supports lymphoma survival via CD40L, BLyS, and IL‐10/STAT3; inhibition triggers apoptosis^58^
*NFATc1*, *NFATc2*, *NFAT5*	Chronic Lymphocytic Leukemia (CLL)	CD23, IL‐10/STAT3 axis, LCK, AQP5–p38 MAPK, anti‐apoptotic pathways	*NFATc2* and *NFAT5* sustain CLL survival by controlling IL‐10/STAT3 signaling and AQP5–p38 MAPK activity	Increased anergy, CLL cell survival and resistance to apoptosis	*NFAT2* is nuclear in CLL, regulating IL‐10/CD23; inhibiting NFAT reverses anergy and reduces survival^58^
NFAT (1–4,5)	Endothelial cells/angiogenesis	VEGF, VEGFR1, β1‐integrin	NFAT regulates VEGF‐driven angiogenesis	Angiogenesis depending on context	NFAT activation enhances VEGF signaling and CsA blocks it^32^

### 3.2 NFAT cross‐talk with signaling pathways

**FIGURE 2:**
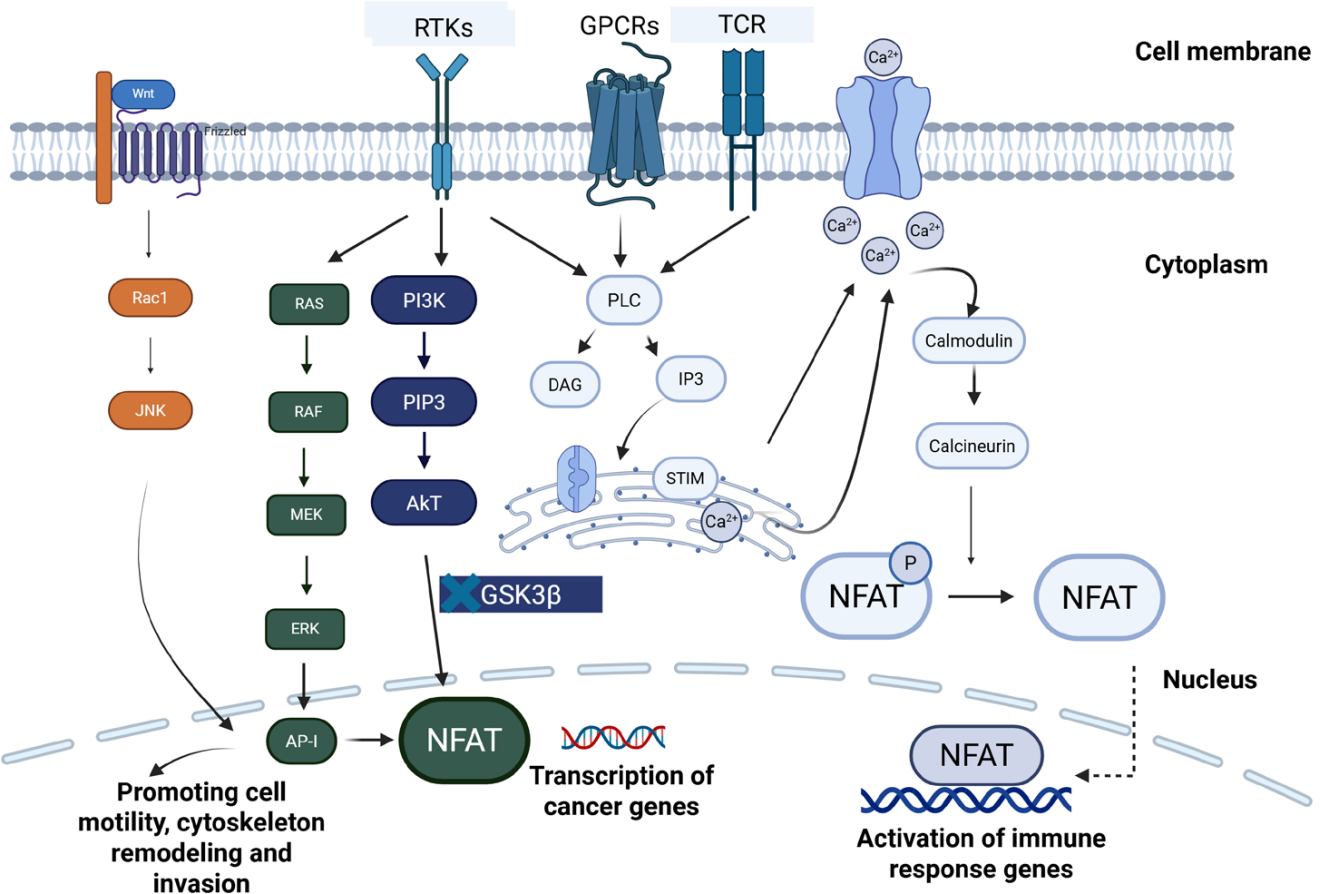
NFAT‐ calcineurin activation and cross‐talk with other signaling pathways. Calcium signaling is integrated in the calcineurin‐NFAT pathway in controlling immune response. Cell surface receptors are activated leading to the cleavage of membrane‐bound phospholipase C (PLC), which splits IP3 and DAG. IP3 causes the release of calcium into the endoplasmic reticulum, which is detected as calcium channels by STIM proteins and keeps the calcium influx. The elevation of the intracellular calcium binding to calmodulin leads to activation of calcineurin phosphatase and subsequently NFAT is dephosphorylated and translocates to the nucleus, where it cooperates with transcription factors in gene transcription. The oncogenic pathways that are responsible in promoting cancer progression are the MAPK and PI3K‐AKT and Wnt pathways. The process starts with the stimulation of small GTPase Ras by RTKs at the plasma membrane. Ras stimulates Raf, MEK and ERK kinase cascade. ERK is activated and translocates to the nucleus where it phosphorylates transcription factors such as AP‐1 (a Fos/Jun complex), which leads to the expression of genes controlling proliferation and invasion. Alongside this, RTKs stimulate PI3K, which phosphorylates PIP2 to produce phosphatidylinositol 3,4,5‐trisphosphate (PIP3). PIP3 binds to the AKT to the membrane and is phosphorylated and activated by PDK1. Activated AKT suppresses the activity of the kinase GSK3β, which normally inactivates NFAT and other substrates through phosphorylation. The inhibition of GSK3β by AKT increases the duration of the action of NFAT within the nucleus, thereby increasing expression of cancer related genes. Frizzled receptors are bound by Wnt ligands on the cell membrane and activate Disheveled (Dvl) proteins. This induces non‐canonical Wnt branches in which Dvl induces small GTPase Rac1. The Rac1 is then activated and prompts JNK (c‐Jun N‐terminal kinase) to phosphorylate and activate transcription factors including AP‐1, a heterodimer of Fos and Jun proteins.

It has been known for some time that NFATs interact with numerous significant signaling pathways that are vital to the development of tumors (shown in Figure 2). The MAPK and PI3K‐AKT cascade is one of them. NFAT activation can occur via RTKs activating such pathways that cause NFAT‐mediated gene expression of cell proliferation, invasion and metastasis. NFAT is known to communicate with the Wnt/β‐catenin pathway and thus it can facilitate the advancement of several malignant traits including tumor growth and angiogenesis.^75^ TGF‐β also has some cross‐talk with NFAT because TGF‐β may regulate NFAT in a Smad‐dependent fashion.^76^ By encouraging invasion, the epithelial‐mesenchymal transition (EMT), and other pro‐tumorigenic processes, this NFAT‐TGF‐β cross‐linkage accelerates the growth of tumors.^77^ Besides the canonical Wnt/β‐catenin pathway, NFAT also interacts with the non‐canonical Wnt signaling pathways, such as the Rac1‐JNK pathway that enhances tumor cell migration, invasion, and other malignant characteristics.^78^ To conclude, NFAT has been discovered to interact with important carcinogenic signaling pathways such as MAPK, PI3K‐AKT, Wnt and TGF‐β. This enables NFAT to promote numerous cancer characteristics such as proliferation, invasion, metastasis and angiogenesis, which facilitates general tumor development.^32^, ^79^

### 3.3 Role in epithelial‐to‐mesenchymal transition and metastasis

NFAT is a chief factor in facilitating epithelial‐to‐mesenchymal transition (EMT) and metastasis, which are major tumor progression drivers.^16^ NFAT activation induces the expression of EMT‐associated genes including N‐cadherin, MMP‐2 and MMP‐9 that enable loss of cell–cell adhesion and acquisition of the migratory and invasive abilities of cancer cells. Moreover, NFAT is able to promote the expression of pro‐angiogenic molecules such as VEGF, which stimulates the development of new blood vessels to sustain tumor development and metastasis. These angiogenic and invasion‐related genes are regulated by NFAT, which adds to the general growth, invasion and metastatic potential of the tumor. Overall, the cross‐talk between NFAT and signaling cascades, including TGF‐β and angiogenic pathways, enables NFAT to be a critical mediator of EMT, invasion and metastasis, which are characteristic of tumor aggression.^23^, ^32^, ^79^

## 4 NFAT IN CANCER STEM CELL BIOLOGY

### 4.1 NFAT regulation and mechanistic roles in cancer stem cell self‐renewal

Cancer stem cells (CSCs), a subpopulation of tumor cells, have the ability to self‐renew, grow, and start tumors. These CSCs have been linked to the development, spread, metastasis, and treatment resistance of tumors. NFAT is essential in controlling the malignant behavior of CSCs by a comprehensive form of cross‐talk among other vital signaling pathways. For instance, the *NFATc2*‐NDEL1‐ERK axis has been demonstrated to stimulate cell multiplication, invasion, and a cancerous phenotype in glioma stem cells.^80^ Similarly, the Ca^2+^/*NFATc2* pathway has been found to play a role in lung cancer regulating stem cell traits, such as tumor growth, cell motility, and resistance to drugs. NFAT interacts with the PI3K‐AKT cascade as well as promoting the resilience to drugs and self‐renewal of CSCs.^71^ The TRPC3‐Ca^2+^‐AKT/GSK‐3β/CNB2‐*NFATc2* axis has been demonstrated to induce the stemness of gastric cancer stem cells in gastric cancer.^81^ Additionally, the resistance of pancreatic cancer stem cells to apoptosis is mediated via the CUX1‐WNT5A‐*NFATc2* axis, which is downstream of the PI3K‐AKT pathway.^82^ NFAT signaling also interacts with Wnt and TGF‐β signaling to maintain stemness and malignant phenotype in CSCs. Similarly, the TGF‐β‐NFAT pathway has been linked to the control of the malignant nature of breast cancer stem cells.^32^

### 4.2 NFAT interaction with stemness pathways

The NFAT transcription factor family maintains CSC properties by interacting with important stemness pathways such as Hippo, NOTCH, and SOX2. For instance, *NFATc2* has been shown to interact with the Hippo‐YAP1 pathway promoting self‐renewal and stemness in colorectal CSCs.^70^ Furthermore, current research suggests that the NFAT‐Notch axis controls tumor development and progression by modulating CSC features.^83^ Additionally, NFAT has been connected to the control of the stemness transcription factor SOX2. It has been demonstrated that *NFATc2* increases SOX2 expression, which leads to the maintenance of gastric CSCs via a similar NFAT‐SOX2 axis and the induction of drug resistance and CSC characteristics in lung adenocarcinoma.^71^ These findings emphasize NFAT's critical function in cross‐talk between NFAT and stemness related pathways, which can contribute to CSC malignancy and tumor growth.

## 5 NFAT'S ROLE IN THE TUMOR IMMUNE MICROENVIRONMENT

### 5.1 Regulation dynamics of T cells

In the tumor microenvironment (TME), NFATs have a pivotal role in controlling T cell activation. In reaction to TCR stimulation, NFAT proteins go to the nucleus, where they activate expression of genes necessary for effector functions. Only tumor‐induced CD8^+^ T cell exhaustion requires *NFAT5*, an NFAT protein without an AP‐1 docking site, which has been shown to be highly expressed in exhausted CD8^+^ T cells during persistent infections and tumors. Due to increased expression of the exhaustion‐associated proteins TOX and PD‐1, which eventually reduce anti‐tumor activity, excessive expression of *NFAT5* in CD8^+^ T cells has been shown to reduce tumor control.^84^ *NFATc2* (NFAT1) can have a role in both cell‐intrinsic tumor suppression and extrinsic tumor promotion, depending on the host parenchyma and tumor microenvironment. *NFATc2* expression is a prospective focus for targeted treatment of some advanced cancers since it can stimulate the proliferation of transformed cells and is probably TGF‐β dependent but independent of IL‐4.^85^

### 5.2 NFAT control of immune checkpoint

NFAT directly regulates the transcription of the checkpoint molecules PD‐1 and CTLA‐4, which are significant inhibitors of T cell activation in cancer. *NFATc1* and *NFATc2* bind directly to the PD‐1 (*Pdcd1*) gene promoter and activate transcription in response to the stimulation of TCR, coupling PD‐1 expression strength to TCR signaling intensity.^86^ When stimulation stops, Blimp‐1 inhibits *NFATc2* and silences PD‐1.^87^ In chronically activated or “exhausted” T cells, *NFATc2* continues to drive PD‐1 production independently of AP‐1, resulting in immunological suppression.^88^ Meanwhile, FoxO1 and BATF promote PD‐1 expression and T cell malfunction, creating a feedback loop that maintains exhaustion over prolonged antigen exposure.^89^

## 6 DYSREGULATED NFAT SIGNALING IN SPECIFIC CANCER TYPES

NFAT signaling exhibits a variety of roles in human cancers, affecting tumor growth through context‐dependent molecular processes.

### 6.1 Gliomas

**FIGURE 3:**
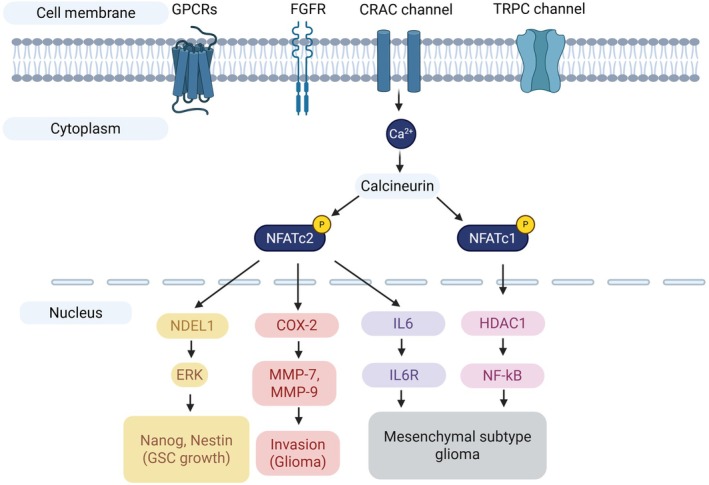
Glioma progression and maintenance of stemness by calcium activated *NFATc1/c2*. The influx of calcium via calcium release‐activated calcium (CRAC) channels causes activation of the phosphatase calcineurin in glioma cells, which dephosphorylates and translocates *NFATc1* and *NFATc2* to the nucleus. In the nucleus, *NFATc1* works together with histone deacetylase1 (HDAC1) to modulate the NF‐kB pathway linked to a mesenchymal subtype of glioma, controlling the expression of genes that cause the aggressive tumor phenotype. *NFATc2* promotes the transcription of targets NDEL1, cyclooxygenase‐2 (COX‐2), and interleukin‐6 (IL‐6), as well as downstream effectors, to stimulate glioma stem cell (GSC) growth, invasion and inflammation. Such pathways incorporate calcium‐calcineurin signaling to suppress glioma progression by mediating the growth and invasion of tumor cells and maintenance of tumor cell phenotype.

Gliomas are malignant tumors of the CNS, marked by rapid invasiveness, neovascularization, and resilience to combination therapy.^90^ NFAT has an important function in the growth and survival of gliomas. *NFATc2* increases the promoter activity of IL6 and IL6R, which contributes to the hostile nature of gliomas. The *NFATc2*‐regulated IL6 signaling pathway facilitates the growth, invasion and tumor initiation of glioma stem cells (GSCs).^91^ Furthermore, by upregulating invasion‐related genes such MMP‐7, MMP‐9, and COX‐2 (shown in Figure 3), *NFATc2* promotes invasion rather than cell proliferation in glioblastoma multiforme, which is the most aggressive kind of glioma.^92^ This suggests that *NFATc2* may be crucial in controlling the malignancy of GSCs. Since APLNR, a receptor associated with advanced gliomas and a poor prognosis, adversely regulates *NFAT5*, it is also involved in the progression of gliomas. The persistence of GSC traits and tumor aggressiveness may be attributed to the deregulation of the NFAT5‐APLNR axis.^93^

### 6.2 Breast cancer

Breast cancer is caused by unregulated epithelial cell proliferation and breast cancer stem cells (BCSCs) have a poorer prognosis.^94^ NFAT regulates a variety of actions in breast cancer cells. The Cn/NFAT pathway was discovered to be active in cases of breast cancer and contributes to the tumorigenicity and progression of breast tumor cell lines.^95^ NFAT may increase the migration of breast cancer cells by activating pro‐metastatic proteins such as COX‐2 and prostaglandin production.^96^ Importantly, NFAT modulates breast cancer angiogenesis. *NFATc3* was discovered as a key facilitator of VEGF‐induced angiogenesis in breast tumors and inhibiting *NFATc3* using the traditional Chinese herb Qingdu granule inhibited tumor angiogenesis.^97^, ^98^ The TGF‐β signaling pathway promotes the oncogenic nature of BCSCs.^99^ In breast cancer, TGF‐β regulates NFAT, which modulates the expression of cell cycle regulators and EMT‐related genes driving tumor growth.^76^, ^77^ Furthermore, the Ca^2+^‐NFAT axis may be critical in retaining BCSC stem cell properties, as calcium signaling has been found to enhance self‐renewal and chemoresistance.^100^

### 6.3 Liver cancer

**FIGURE 4:**
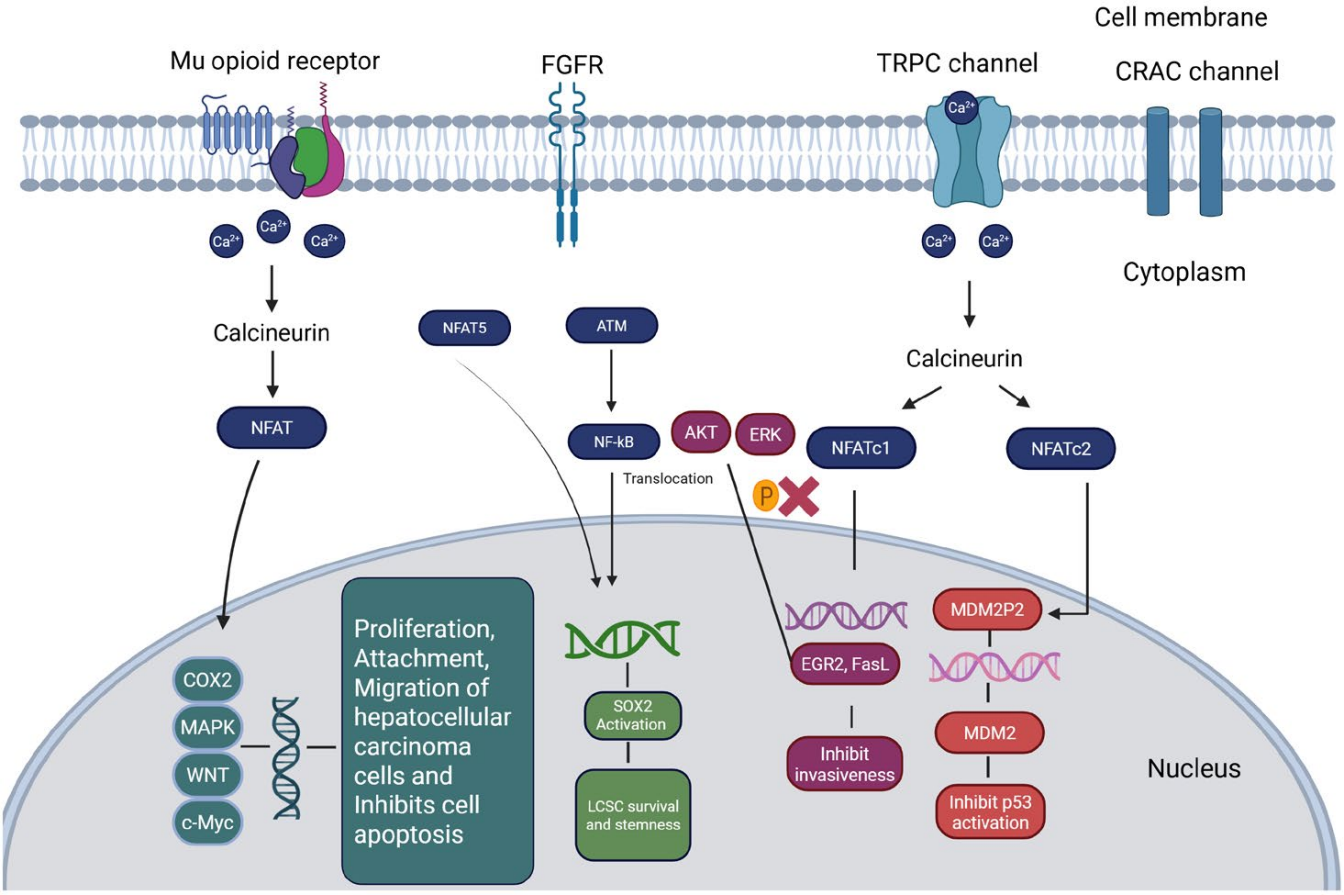
NFAT signaling of hepatic cancer promotes stemness, growth, and metastasis. NFAT signaling enhances proliferation, migration and survival of liver cancer stem cells (LCSCs) by transcriptionally activating oncogenic targets such as MDM2 via *NFATc2*, which suppresses p53. *NFATc1* causes apoptosis and inhibits invasion by Egr2 and FasL. The MOR‐NFAT pathway also controls HCC proliferation, migration and self‐renewal via MAPK, c‐Myc, WNT and COX2 pathways. NFAT5 also triggers the ATM‐NF‐kB‐SOX2 pathway to stimulate LCSC self‐renewal, which disrupts tumor remission and leads to an unfavorable prognosis.

Liver cancer is a very common cancer with a relatively high mortality rate. Treatment resistance, metastasis, and tumor recurrence are all associated with liver cancer stem cells (LCSCs).^101^ NFAT promotes the development of liver cancer in a number of ways. *NFATc2* inhibits p53 activation while promoting the growth of liver cancer cells via binding to the MDM2 promoter and activating it through transcription in a p53‐independent way.^102^ However, it has also been shown that *NFATc1* increases the expression of FasL and Egr2, which reduces the invasiveness of liver cancer cells and accelerates their apoptosis.^103^ Hepatocellular carcinoma (HCC) tissues and cell lines exhibit downregulated NFAT5, whereas its overexpression results in apoptosis and inhibits cellular invasion. Nonetheless, it has been demonstrated that the MOR‐NFAT axis improves the proliferation, migration, and self‐renewal of hepatocellular carcinoma stem cells,^104^ and that the FGF19/FGFR4‐SOCE/*NFATc2* signaling pathway is necessary for LCSC self‐renewal.^105^ Furthermore, *NFAT5* starts ATM‐regulated NF‐kB activation, which boosts LCSC stemness and self‐renewal through the ATM‐NF‐kB‐SOX2^55^ axis (shown in Figure 4).

### 6.4 Colorectal cancer

Colorectal cancer stem cells (CCSCs) play a part in tumor resistance and recurrence. Colorectal cancer is the third most common cause of cancer‐related death worldwide.^106^ Both the growth of colorectal cancer and the creation of CCSCs are encouraged by NFAT proteins. Colorectal cancer cells that were exposed to acid expressed more of the acid‐sensitive ion channel ASIC2, which activated the calcineurin/*NFATc2* pathway and promoted the growth, invasion and metastasis of CRC cells to the liver.^69^ According to a recent study, the NFAT and Hippo‐YAP1 pathways cooperate and the Hippo‐YAP1 signaling pathway is essential for maintaining CCSC stemness. *NFATc2* specifically inhibits YAP phosphorylation, hence increasing its transcriptional activity and enhancing CCSC stemness.^70^ The calcium route is also critical for CCSC promotion as epithelial calcineurin increases CCSC survival and proliferation through NFAT.^107^ Additionally, the *NFAT5*‐JMJD2B‐NANOG axis was discovered to regulate dedifferentiation and stemness in colorectal cancer.^72^

### 6.5 Squamous cell carcinoma

*NFAT5* is a crucial factor for accelerating oral cancer cell growth. *NFAT5* exerts this function by upregulating DPAGT1 expression which facilitates the translocation of EGFR to the plasma membrane from the endoplasmic reticulum. DPAGT1 activation and EGFR subcellular translocation, which are linked with OSCC tumor progress, depend on hypertonic‐induced *NFAT5* translocation.^108^ In oral squamous cell carcinoma, prolonged ethanol exposure stimulates the NFAT signaling system, which increases CSC populations and stem cell characteristics like ALDH activity, self‐renewal ability, stem cell marker expression and migratory potential.^109^ Orai stimulates *NFATc3*, which enhances OCT4 expression by increasing OCT4 promoter activity. The *NFATc3*‐OCT4 axis enhances stem cell survival and self‐renewal in oral squamous cell cancer.^110^

### 6.6 Lung cancer

Lung cancer development is significantly influenced by the NFAT family. As2O3 has been shown to prevent SCLC metastasis and endothelial cell activation through the calcineurin NFAT signaling pathway. *NFATc2* was shown to be highly expressed in lung tumor tissues, and *NFATc2* knockdown dramatically decreased lung cancer cell invasion, migration, and proliferation.^111^ Furthermore, it was shown that *NFAT5* modulates the expression of AQP5, which in turn influences the proliferation and migration of human lung adenocarcinoma cells.^73^ Anomalies in calcium signaling may contribute to the malignant nature of lung cancer stem cells (CSCs). Voltage‐gated calcium channel α2δ1‐expressing SCLC cells exhibited CSC traits and were treatment‐resistant.^112^ The Ca^2+^/NFATc2 pathway is one novel regulator of lung CSC properties, including tumor sphere formation, cell mobility, carcinogenesis, and therapeutic response. Furthermore, CSC traits and treatment resistance in lung cancer are caused by the *NFATc2/SOX2/ALDHA1* axis, and *NFATc2* enhances SOX2 expression via its 3′ enhancer. In conclusion, via regulating lung CSCs, the NFAT family plays a major role in the development of the malignant characteristics of lung cancer cells, including their invasiveness, ability to spread, and resistance to treatment.^71^

### 6.7 Gastric cancer

A malignant transformation of the gastric epithelium is termed gastric cancer (GC). Aberrant activation of calcium signaling increases the survival of gastric cancer stem cells (GCSCs). TRPC3 enhances Ca^2+^ influx and induces the carcinogenic nuclear translocation of *NFATc2* in gastric cancer cells.^81^ Blocking the *STAT3/MSK1/NFATc2* signaling axis has been found to prevent the multiplication of gastric cancer cells as well as the formation of xenogeneic tumors. This shows that aberrant epigenetic and transcriptional pathways involving NFAT could be used to produce new targets for the treatment of gastric cancer.^113^ *NFATc1* participates in cell control and epithelial mesenchymal transition (EMT) programming by creating a complex with SOX2 that is attracted to the Snai1 promoter by the collagen‐bound receptor tyrosine kinase DDR2. This mechanism contributes to the maintenance of gastric CSC stemness.^114^ In summary, the NFAT signaling system, notably the activation of *NFATc2* and *NFATc1*, contributes to the malignant phenotype of gastric cancer cells while also maintaining the stemness of GCSCs. Targeting NFAT‐mediated transcriptional networks may have therapeutic benefits in gastric cancer.

### 6.8 Pancreatic cancer

An extremely aggressive form of malignancy with a dismal prognosis is pancreatic cancer. The inhibition of NFAT promotor causes *NFATc2* to silence p15INK4b and stimulates the growth of pancreatic cancer tumors both in vitro and in vivo. Targeting the calcineurin‐*NFATc2* pathway may be therapeutic for pancreatic cancer since *NFATc2* inactivation has been shown to hamper the disease's progression.^115^ Intracellular calcium signals that trigger the CaM/NFAT pathway cause pancreatic cancer cells to divide and multiply.^116^ One important mechanism for C‐MYC activation in pancreatic cancer is the stimulation of the *NFATc1* and Ca^2+^/CaM signaling pathways. *NFATc1* has been demonstrated to bind to C‐MYC and increase its transcription in pancreatic cancer cells. This *NFATc1*‐mediated C‐MYC overexpression increases the malignant phenotype of pancreatic cancer stem cells (PCSCs) including proliferation, stemness and EMT.^29^ Furthermore, *NFATc1* has been shown to coordinate the expression of EMT‐related genes and stem factors in a SOX2‐dependent way, hence a stem‐like state in pancreatic cancer cells is maintained. This emphasizes the importance of the NFAT‐SOX2 axis in controlling the malignant transformation and stemness of PCSCs.^117^ In conclusion, the NFAT family, notably *NFATc2* and *NFATc1*, plays a pivotal part in accelerating the proliferation, tumor growth, and maintenance of the stem‐like phenotype in pancreatic cancer, making it a prospective therapeutic target in this aggressive malignancy.

## 7 THERAPEUTIC TARGETING OF NFAT

### 7.1 Pharmacological strategies to inhibit NFAT signaling

There are three approaches to targeting the NFAT signaling pathway as a potential cancer therapy. (1) Targeting Upstream Regulators of NFAT. Drugs such as cyclosporin A (CsA), tacrolimus (FK‐506) and ISA247 inhibit the calcineurin phosphatase that dephosphorylates and activates NFAT. There are also other inhibitors such as BTP2 and McKeon compounds that inhibit calcium signaling upstream of NFAT. (2) Directly targeting NFAT. Compounds such as ST‐1959, Roc‐1/2/3, helenalin, genistein and zoledronic acid have a direct effect on inhibiting the expression of NFAT and its nuclear translocation or promoting its degradation. (3) Blocking binding of NFAT to DNA. UR‐1505, triflusal, caffeic acid phenethyl ester (CAPE), punicalagin, and vitamin D3 are some of the compounds that prevent the binding of NFAT to its target DNA sequences, thereby interfering with its transcriptional activity. These approaches will inhibit the NFAT signaling axis that plays a role in many cancer hallmarks such as cell number expansion, invasion, metastasis and angiogenesis.^22^

### 7.2 Preclinical and clinical evidence across cancer types

NFAT plays a multifaceted role in cancer progression and responses to therapy by controlling tumor growth, immune evasion, drug resistance and metastatic behavior via the Ca^2+^‐calcineurin‐NFAT signaling pathway. InuA‐mediated suppression of *NFATc2* prevents MDM2 transcription and prostate cancer proliferation, migration and invasion regardless of p53 or androgen receptor status.^118^ *NFATc2* enhances immune evasion by stimulating PD‐L1 in renal cell carcinoma and is maintained in sunitinib resistant tumors through overactivation of PI3K/AKT/GSK3β. In addition, FOXA1 and SETD2 suppress its degradation through inhibition of FBW7.^119^ Triterpenoids block ASIC2 and *NFATc2* in colorectal cancer, blocking downstream MMP‐2/MMP‐9 expression and invasion. *NFATc2* nuclear translocation and Fas/FasL are increased with combined TMZ and lithium treatment to repress the growth of glioma.^120^ In contrast, *NFATc1* promotes proliferation and prevents DNA damage, thereby mediating the resistance to carboplatin in lung cancer, and indeed, its silencing can restore drug susceptibility of high glucose‐induced lung cancer, which underscores its clinical application in diabetic non‐small cell lung cancer (NSCLC).^121^ miR‐194 regulates the progression of lung cancer by targeting *NFAT5* and predicts its treatment of diabetic NSCLC.^122^ *NFAT5* is also highly expressed in inflammatory breast cancer, where it is a biomarker of aggressive subtypes, and a combination of NFAT expression and CA72‐4 levels predicts the prognosis in ovarian clear cell carcinoma through the regulation of CHAD. Also, AM404, an acetaminophen metabolite, suppresses NFAT and COX‐2 expression in neuroblastoma, which may indicate the possibility of its use as an anti‐inflammatory and anti‐cancer agent.^123^, ^124^ Table 2 summarizes a brief overview of the types of cancer, NFAT‐targeting approaches and the mechanistic consequences of these approaches.

**TABLE 2:** Cancer specific targeting strategies and mechanistic outcomes of NFAT pathway inhibition.

Cancer type	Therapeutic targeting strategy	Key findings and mechanisms
Glioma	Calcineurin inhibitors (CsA, FK506); *NFATc2*‐NDEL1 axis targeting	Inhibition of *NFATc2* reduces glioma invasiveness and tumor growth by suppressing IL‐6 signaling and invasion‐related genes. NFAT‐NDEL1 axis is a promising target^32^
Breast cancer	NFAT inhibitors	*NFATc1* promotes EMT and metastasis through transcriptional regulation of invasion genes like ADAMTS1^95^
Liver cancer	Targeting *NFATc2*‐activated FGF19/SOCE pathway	Blocking *NFATc2* signaling diminishes CSC self‐renewal and tumor progression in hepatocellular carcinoma^105^
Colorectal cancer	*NFATc2* inhibition; YAP1 pathway co‐targeting	*NFATc2* drives CSC stemness and metastasis; co‐targeting Hippo‐YAP1 may improve therapeutic efficacy^70^
Lung cancer	Modulation of NFAT isoforms; immune checkpoint therapy synergy	NFAT isoforms correlate with prognosis; NFAT1 deficiency linked to immune evasion; NFAT targeting could enhance immunotherapy^125^
Gastric cancer	Targeting TRPC3‐Ca^2+^‐AKT/GSK3β‐*NFATc2* axis	Disruption of the pathway reduces CSC proliferation and chemoresistance^81^
Pancreatic cancer	*NFATc1/SMAD3/cJUN* transcription complex disruption; combined targeted therapies	Disruption of the oncogenic *NFATc1/SMAD3/cJUN* transcription factor complex to reduce tumor proliferation and chemoresistance^126^

### 7.3 Bridging spatial transcriptomics and NFAT signaling for precision oncology

Spatial transcriptomics (ST) has proven to be a revolutionary technology to analyze the architecture of tumor microenvironments, cell–cell interactions, immune checkpoint landscapes, and transcriptional heterogeneity in multiple types of cancer, such as lung, colorectal, gastric, breast, and brain tumors.^127^, ^128^, ^129^ ST has revealed significant information about the TME, macrophage reprogramming, immune checkpoints and immunosuppressive alteration in brain metastases in lung cancer, which may serve as prognostic biomarkers and treatment options for immune and fibrotic pathways.^130^, ^131^ In colorectal cancer, ST has assisted in a study of genetic and epigenetic co‐evolution that has shown the effects of chromatin and transcription factor changes on tumor progression and metastasis.^132^, ^133^ The TME cellular components and interactions between them play a significant role in tumor progression, metastasis and prognosis, and are possible new targets of precision therapy.^134^ In gastric cancer, ST can be utilized to investigate the biological characteristics of the tumors of the gastrointestinal tract using the various levels of tissues, identify the differences of gene expression between tumor and the surrounding normal tissues, and obtain valuable information about the early diagnosis of the tumor and predict the recurrence in the tumor.^135^ However, in spite of these recent developments in spatially mapping transcriptional factors and cell ecosystems, there are still no studies that have used ST to comprehensively map NFAT isoform distribution across different cell types and their spatial locations in tumors, which is an urgent gap since NFAT family members have specific, often opposite roles in different cell types, with some isoforms inducting tumors and others repressing tumor progression. The exploitation of ST platforms that have been able to map immune and metabolic programs to decode the complexity of NFAT is an important next step in creating isoform‐selective, spatially‐informed therapeutic approaches that can bypass the limitations of pan‐NFAT inhibitors, which have historically failed due to severe immunosuppression because of their inability to differentiate between beneficial and adverse NFAT functions in various cellular compartments.

## 8 CONCLUSION

The NFAT transcription factor family was initially discovered in T cells and shown to be important in the regulation of the immune response. Later research has demonstrated that NFAT proteins are ubiquitously expressed and have a wide range of regulatory functions in most types of cells, including those implicated in the development of cancer. The NFAT transcription factors have been implicated in regulating extensive variety of cellular functions that are essential in cancer such as in survival of cells, cell growth, cell migration, cell invasion, and angiogenesis. Notably, NFAT isoforms are often constitutively engaged and/or overexpressed in diverse human malignancies, leading to malignant phenotypes and tumor development. Considering the central role of NFAT in cancer, the pharmacological or genetic targeting of NFAT signaling pathway has been proposed as a promising approach to cancer prevention and treatment. Moreover, NFAT inhibitors of various classes have shown promising anti‐cancer effects in preclinical research.

Even with substantial progress, there are still important gaps in our knowledge of NFAT signaling in cancer that should be addressed in the future. To determine context‐dependent functions of individual NFAT members in the tumor microenvironment, isoform and cell type‐specific NFAT mouse models especially focused on tumor‐associated fibroblasts, macrophages, endothelial cells and regulatory T cells will need to be developed. Moreover, although spatial transcriptomics has been successfully applied to map immune checkpoint landscapes, metabolic programs, and transcription factor activities in a variety of cancer types, no research has so far be done to profile systematically NFAT isoform distribution using spatial technologies. Spatial resolution may provide important insights into NFAT biology since within a cellular and spatial context NFAT isoforms often have opposed functions, which cannot be determined by bulk RNA sequencing or traditional immunohistochemistry. Combining spatial transcriptomics with NFAT biology could help clarify which isoforms contribute to oncogenic and tumor suppressive programs in particular tumor areas and cellular subsets to inform the design of spatially targeted therapeutic interventions. The existing pharmacological inhibitors of calcineurin‐NFAT, for example, cyclosporin A and FK506, are not isoform‐selective but instead cause systemic immunosuppression, which highlights the need for more selective approaches, for example, isoform‐specific small molecules, peptide disruptors, or PROTAC‐based degraders. Moreover, despite the evidence of NFAT being involved in immune evasion and regulation of PD‐L1, its contribution to immunotherapy resistance to immune checkpoint blockade and CAR‐T therapy has not yet been fully studied and no clinical trials are currently available that have attempted to combine NFAT targeting with immunotherapy. Although NFAT plays a role in cancer stem cell maintenance in a variety of malignancies, downstream transcriptional programs that regulate self‐renewal and chemoresistance have yet to be clearly determined. Moreover, a diagnostic or prognostic biomarker of NFAT5 and combined NFAT‐CA72‐4 has yet to be clinically validated at large scales and detection of NFAT signatures using liquid biopsy is not available. Together, filling these gaps will be important for improving the translational understanding of NFAT biology to achieve effective and accurate cancer therapeutics.

## AUTHOR CONTRIBUTIONS

**Hira Khan:** Formal analysis; investigation; methodology; writing – original draft; writing – review and editing. **Khushbakhat Alia:** Data curation; formal analysis; methodology; software; visualization; writing – review and editing. **Yusra Al Dhaheri:** Data curation; investigation; methodology; visualization; writing – review and editing. **Muhammad Naseem:** Data curation; formal analysis; methodology; visualization; writing – review and editing. **Rabah Iratni:** Data curation; formal analysis; methodology; writing – review and editing. **Edgar Serfling:** Formal analysis; investigation; validation; writing – review and editing. **Khalid Muhammad:** Conceptualization; formal analysis; funding acquisition; project administration; resources; supervision; writing – original draft; writing – review and editing.

## FUNDING INFORMATION

This work is supported by UAE University grants (UPAR grant G‐4960, UAEU‐ZU joint research grant G‐5310 and Zayed center for health sciences grant G‐5500) to K.M.

## CONFLICT OF INTEREST STATEMENT

The authors declare that they have no known competing financial interests or personal relationships that could have appeared to influence the work reported in this paper.

## ETHICS STATEMENT

Not applicable. This article is a review and does not contain any new studies involving human participants or animals performed by the authors.

## GENERATIVE AI STATEMENT

The authors declared that generative AI was not used in the creation of this manuscript.

## Data Availability

None.
